# Effects of Tobacco Smoking on the Survivability of Patients with Multiple Cancers and Single Lung Cancer

**DOI:** 10.3390/ijerph19159179

**Published:** 2022-07-27

**Authors:** Anna Romaszko-Wojtowicz, Andżelika Lorenc, Adam Buciński, Anna Doboszyńska

**Affiliations:** 1 Faculty of Health Sciences, Department of Pulmonology, University of Warmia and Mazury in Olsztyn, 10-357 Olsztyn, Poland; anna.doboszynska@wp.pl; 2 Department of Biopharmacy, Ludwik Rydygier Collegium Medicum in Bydgoszcz, Nicolaus Copernicus University in Toruń, Jurasza 2, 85-089 Bydgoszcz, Poland; andzelika.lorenc@cm.umk.pl (A.L.); adam.bucinski@cm.umk.pl (A.B.)

**Keywords:** lung cancer, multiple neoplasm, tobacco smoking

## Abstract

Lung cancer is the leading cause of death worldwide among men and women. Tobacco smoking is the number one risk factor for lung cancer. The aim of our study was to evaluate the survivability of patients with single lung cancer in relation to the survival time in patients with multiple neoplasms whose last neoplasm was a lung cancer. A retrospective analysis was con-ducted of data from medical histories of patients hospitalized at the Pulmonary Hospital in Olsztyn (Poland) from 2012 to 2017, with a lung cancer diagnosis as the first or subsequent cancer. The total longevity of women with diagnosed multiple cancers was found to be shorter than that of men: 67.60 years (SD: 7.77) and 69.91 years (SD: 7.97), respectively. Among the ex-smokers, the longevity of men (68.93 years) was longer than that of women (66.18 years). Survival time, counted from the diagnosis of both the first and subsequent cancer, was longer among patients with multiple cancers than among patients with single lung cancer (*p* = 0.000). Women’s survivability was worse than men’s in the case of multiple cancers and in the group of people who quit smoking (*p* = 0.037; *p* = 0.000). To conclude, smoking tobacco affects the survival of patients with lung cancer. Smoking cessation improves overall survival.

## 1. Introduction

Lung cancer is one of the most frequently diagnosed neoplasms and the main cause of death due to neoplasms globally, among both men and women [1]. Every year, around 2 million new cases are diagnosed and 1.76 million people die [2]. Most patients are diagnosed in an advanced stage, with fewer than 20% of cases diagnosed early enough for surgical treatment [3]. The development of medical technologies and progress in diagnostic and therapeutic methods as regards cancer patients contribute to a better prognosis and prolong survival time. However, this development is increasingly often associated with the secondary development of new malignancies.

Patients with a second or even third neoplasm constitute an ever-growing population that requires special attention. Primary multiple neoplasms develop in their location and are not part of an existing neoplastic process, nor are they spread, metastasized, or recurrent [4].

The development of another neoplastic process is influenced by a combination of genetic and external factors, for example, environmental or occupational circumstances or previous oncological treatment. The group of environmental factors increasing the risk of oncogenesis includes: ultraviolet radiation, atmospheric pollution, e.g., diesel exhaust, as well as some viral infections (EBV, HPV, HBV, HCV), obesity, smoking, and alcohol consumption [5,6]. Pesticides, industrial and production chemicals, and aflatoxins in the work environment contribute to an increased risk of lung cancer development. Agricultural workers are exposed to the effects of chemicals, such as chlorimuron-ethyl and parathion, which are directly associated with lung cancer development [7,8]. Occupational exposure has also been related to welder fumes among industrial workers [9,10].

The data obtained from many scientific studies confirm the direct influence of both active and passive smoking on the development of lung cancer [6]. According to the 2019 Report the Chief Sanitary Inspectorate, one in five adults smoke in Poland [11]. The popularity of smoking varies depending, among others, on socio-economic factors. In 2010, the so-called anti-smoking act banned smoking in public places, undoubtedly contributing to the downward tobacco smoking trend. Nevertheless, smoking remains the primary determinant of lung cancer incidence. Tobacco smoking has a multi-faceted impact on lung cancer [12,13,14]. On the one hand, tobacco smoking is a known risk factor in the development of lung cancer, and is associated with worse survivability and a higher risk of developing a subsequent cancer. Furthermore, there are many contradictory reports in the literature concerning the survivability of patients who have ceased smoking. Components of tobacco smoke lead to lung barrier dysfunction and a chronic inflammatory state resulting in epithelial-mesenchymal transmission and subsequent tissue remodeling. Moreover, carcinogens (PAH, N-nitrosamines, aromatic amines, 1,3-butadiene, benzene, aldehydes, etc.) in tobacco smoke induce the oncogenesis process by damaging DNA. They also play a significant role in disrupting the balance between the mechanisms leading to or inhibiting apoptosis. Gene mutations can cause losses of normal cell growth control functions, eventually leading to cell proliferation and cancer. The reversibility of cancer risk after smoking cessation supports the role of tumor promoters and other epigenetic factors in tobacco carcinogenesis [15]. Smoking cessation before lung cancer diagnosis reduces the risk of primary lung cancer development of all major histopathological types of lung cancer. The most significant reduction in inflammation is seen in small and squamous cell carcinomas [16]. Shiels et al. showed that smoking before the first cancer diagnosis increases the risk of developing another cancer process in those patients who survive [17]. Romaszko et al. in 2018 showed that the discontinuation of smoking after the first cancer has been diagnosed lengthens the survival time of patients with multiple cancers compared to patients who continue to smoke [18]. In 2008, Ozasa et al. demonstrated that the survival time of patients who stopped smoking was longer than that of those who continued to smoke, and in terms of sex division, women lived longer than men [19]. However, some patients continue to smoke despite a lung cancer diagnosis or resume the addiction. Therefore, it is purposeful to conduct research on a group of patients with multiple neoplasms, the incidence of which is increasing. In a previously published study, we found that among patients with multiple cancers, those who never smoked lived longer than those who continued smoking, and those who quit smoking lived longer than those who continued smoking. Aredo et al., in a meta-analysis conducted on over 7000 patients, showed that smoking is a risk factor for the development of second primary lung cancer, and quitting smoking may reduce this risk [20].

For the present study, we considered the influence of environmental factors, which include all occupational exposures and, in particular, smoking. The aim of our study was to evaluate the survivability of patients with single lung cancer in relation to the survival time in patients with multiple neoplasms whose last neoplasm was lung cancer.

Particular emphasis in the paper was placed on the influence of environmental factors, especially the impact of smoking.

The following hypotheses were put forward in the article:

1. Smoking is an important factor in the development of neoplastic diseases and affects the survival of patients.
2. Smoking cessation contributes to extending the life span of patients.
3. The patients’ survival depends on the risk factors, including the previous treatment of the neoplastic disease.
4. Patients with multiple cancers whose last cancer process was lung cancer live longer than patients with single lung cancer.

## 2. Materials and Methods

### 2.1. Patient and Study Design

The Warmińsko-Mazurskie Centre for Pulmonary Diseases is a reference pulmonological center in north-eastern Poland. A large proportion of patients with changes in lungs suspected to be neoplastic in character are hospitalized in this hospital.

Among all hospitalized patients from the beginning of 2012 to the end of 2017, 2321 patients were identified with the diagnosis D38.1 (according to ICD10). The inclusion criterion was a histopathologically confirmed diagnosis of lung cancer—in total, 2301 persons (2079 with a single cancer and 222 with multiple cancers). The exclusion criterion was a lack of a histopathological diagnosis for various reasons (e.g., premature death of the patient) or a diagnosis of disseminated neoplastic disease with a point of origin outside the lung. The adopted time criteria were based on the availability of medical data from the IT system and the assumption of a 5-year observation period for patient survival.

### 2.2. Data Collection

The study consisted of a retrospective analysis of data collected from medical histories of patients hospitalized in the Warmińsko-Mazurskie Centre for Pulmonary Diseases in Olsztyn, having obtained the consent of the Centre’s authorities and the Bioethics Committee. The patients were divided into two groups: those with a single cancer and those with multiple cancers. Multiple cancers were identified according to the IARC definition. In line with the time criterion (6 months), patients with synchronous and metachronous cancers were distinguished [21]. The following epidemiological data were collected: tobacco smoking, exposure to hazardous environmental factors and family history. According to the literature, a non-smoker is a person who has smoked no more than 100 cigarettes in their lifetime, and an ex-smoker is someone who has not smoked for at least 6 months [22,23]. Family history was considered to be positive if a neoplastic disease occurred in first-degree relatives (parents, brothers, sisters, daughters, or sons). Total survival time was calculated from the date of diagnosing primary cancer to the date of death or the last observation. The information about the date of death was obtained from the Central Statistical Office. In accordance with the principles of Good Clinical Practice, obtaining information about regarding patients’ deaths was preceded by the consent of the Bioethics Committee. The processing and storage of data complied with the applicable GDPR directive. At the University of Warmia and Mazury in Olsztyn, personal data are protected at all levels. Existing procedures were applied to the project. All patients were given study ID numbers and patient data were anonymized.

### 2.3. Statistical Analysis

The statistical analysis was conducted in Statistica 13.3 (StatSoft Inc., Tulsa, OK, USA). The mean, median and standard deviations were determined for the analyzed data. The normality of the distribution of variables was verified with the W Shapiro–Wilk test, and the equality of variances in groups was assessed with Levene’s test. Differences between the groups of the variables with a normal distribution and equality of variances were determined with the parametric t-Student test (for two groups) or the ANOVA with LSD post-hoc analysis (for more than two groups). The variables whose distribution deviated from normal or which did not reveal an equality of variances were submitted to the non-parametric U Mann–Whitney test or ANOVA Kruskal–Wallis with a two-sided multiple comparison test, respectively. The survival time of patients was analyzed using the Kaplan–Meier test. The Pearson chi-square test was used to compare the number of qualitative variables. The statistical significance level for all the tests was set at *p* < 0.05.

Dependent variables, namely, packyears, age of 1st cancer, age of 2nd cancer, interval between 1st and 2nd cancer, general total survival time in years, smokers’, ex-smokers’, and non-smokers’ total survival time in years, and survival time since 1st cancer (years), were tested in relation to gender, single and multiple neoplasms, and tobacco intake status (independent variables).

### 2.4. Ethics Statement

The study is a retrospective analysis of data from the medical histories of patients, such as date of birth, date of death, sex, age, comorbidities, and risk factors of developing cancer. No personal data of patients were processed. The study was issued a positive opinion by the Bioethics Committee at the Nicolas Copernicus University in Toruń, Collegium Medicum in Bydgoszcz, on 23 June 2020 (KB 355/2020).

## 3. Results

The retrospective analysis of data comprised 2321 medical histories, including histories of 2301 persons (724 women and 1577 men) with a histopathologically confirmed diagnosis of a malignant lung neoplasm. A total of 2079 patients had a single cancer diagnosed, while 222 patients had multiple cancers diagnosed of which the last neoplastic growth was of lung cancer. The first primary cancers in patients with multiple neoplasms were: leukemia, lymphoma, head and neck cancer, ovaries cancer, uterus cancer, kidney cancer, bladder cancer, breast cancer, prostate cancer, colon cancer, sarcoma, skin cancer, thyroid cancer, Hodgkin cancer, and stomach cancer. More detailed information about the patients is shown in Table 1.

The Pearson chi-square test showed significant differences in the number of non-smoking patients of both sexes in the whole group (*p* = 0.000) and the subgroups of patients with a single tumor (*p* = 0.000) and multiple neoplasms (*p* = 0.002). Significant statistical differences were also observed between the number of patients with single tumors compared to patients with multiple neoplasms in all three groups related to tobacco intake.

**Figure 1:**
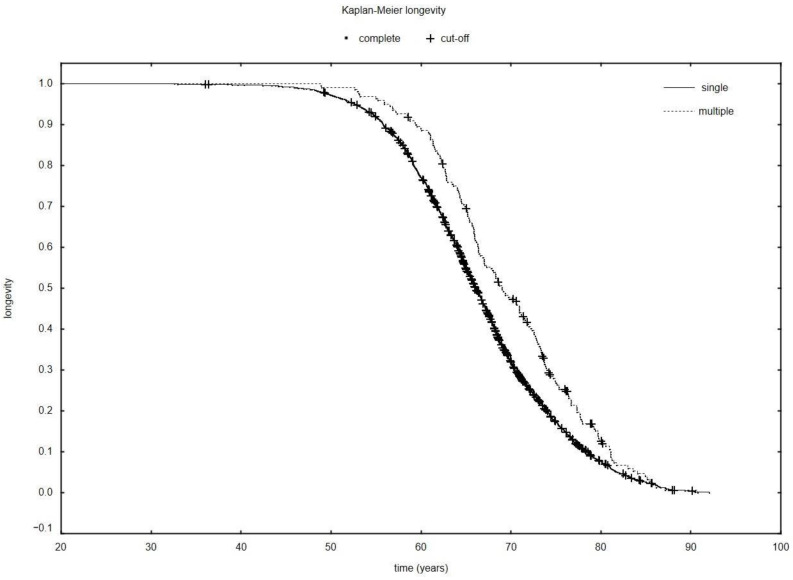
The Kaplan–Meier longevity in patients with multiple and single neoplasms.

Based on the collected data, it was demonstrated that the survival time of patients with single lung cancer (1.31 years; SD: 1.48) was considerably shorter than that of patients with multiple neoplasms (8.02 years; SD 7.52) (*p* = 0.000) counted from the diagnosis of the first cancer (Figure 1) as well as total survival time (single: 65.80 years; SD: 8.39; multiple: 69.18 years SD: 7.96) (*p* = 0.000). The exact data on the survival of the patients are presented in Table 2.

**Figure 2:**
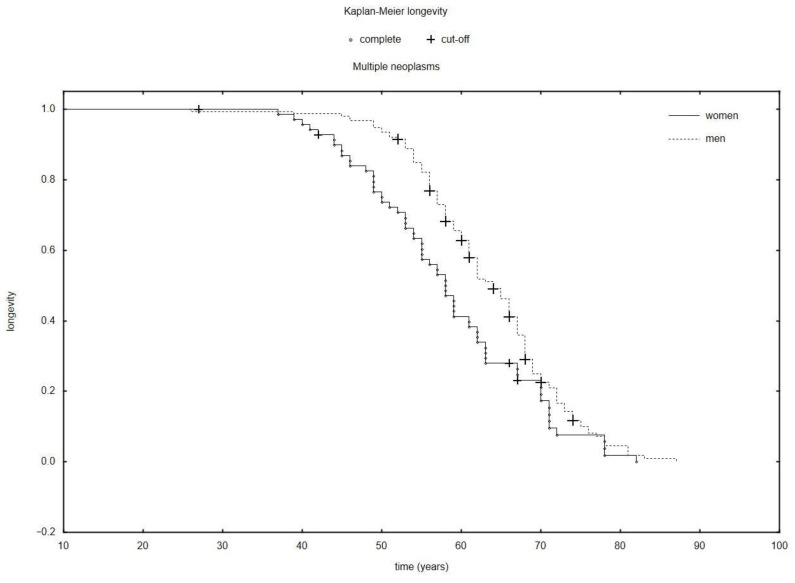
The Kaplan–Meier longevity in a population of women and men with multiple neoplasms.

The research shows that the total longevity of women with a diagnosis of multiple neoplasms was shorter than that of men: 67.60 years (SD: 7.77) versus 69.91 years (SD: 7.97); *p* = 0.037 (Figure 2). However, it is worth underlining that the survivability since the diagnosis of the first neoplasm was statistically longer for women than for men: 10.19 years (SD: 8.93) versus 7.02 years (SD: 6.56); *p* = 0.01. Interestingly, we also demonstrated that the time elapsing until the development of a subsequent neoplasm was demonstrably longer in women than in men: 8.79 years (SD: 8.88) versus 5.76 years (6.52); *p* = 0.015.

In the group of smokers, also having included the division into the groups of women and men, the patients with single neoplasms (64.78 years; SD: 7.97) lived statistically significantly shorter lives than patients with multiple neoplasms (66.74 years; SD: 7.77) (*p* = 0.014).

**Figure 3:**
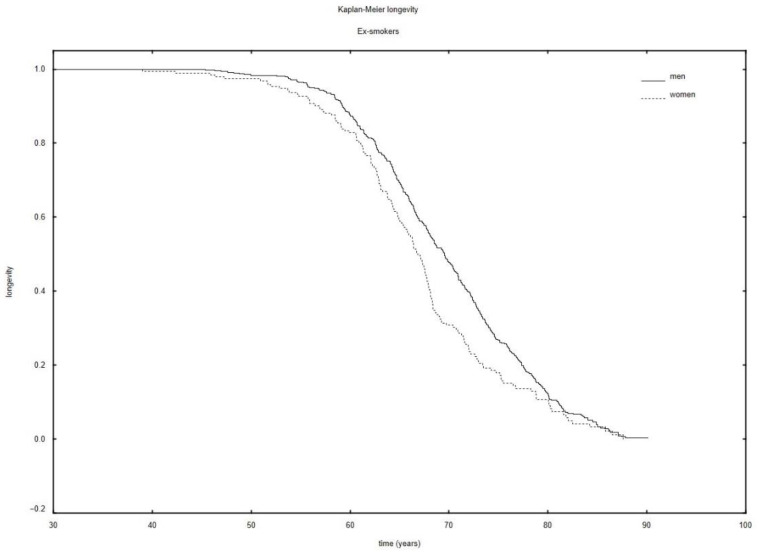
The Kaplan–Meier longevity in ex-smokers.

It was also shown, in the group of patients with multiple neoplasms, that men had a statistically significantly longer survival time than women (72.32 years; SD: 7.11 vs. 66.82 years; SD: 6.11) (*p* = 0.001) among ex-smokers. These data are illustrated in Figure 3. Nevertheless, survival time since first cancer diagnosis was longer for women than for men (10.19 years; SD: 8.93 vs. 7.02 years; SD: 6.56) (*p* = 0.010).

Analysis of variance (ANOVA) showed statistically significant differences (*p* < 0.05) in total survival time between groups of smokers, ex-smokers, and non-smokers, taking into consideration gender and single/multiple neoplasms. The results of the performed tests are shown in Table 3.

## 4. Discussion

Most of the analyzed cases (n = 2174; 94.48%) were patients with a history of tobacco addiction. In this group, there were 65.01% (n = 1496) smokers and 29.46% (n = 678) people who quit smoking (Table 1). In our study, we demonstrated a positive effect of quitting smoking on the overall survival of patients in the group of women and that of men. A statistically significant improvement was not observed only in women with multiple cancer groups. Moreover, the study showed that men live longer than women among people who quit smoking, broken down by gender (*p* = 0.000) (Table 2 and Table 3). Although historically lung cancer used to be more frequent among men than women, the proportions of men and women diagnosed with this illness have changed rather drastically over the past twenty years. The incidence of lung cancer has been decreasing among men for several decades now, whereas in most countries around the world there has been an increasing tendency for many years now for women to develop this type of cancer [24,25,26]. It is estimated that morbidity decreases approximately twice as rapidly among men than among women, which reflects the delay in women quitting smoking observed in society over time [3,27].

For the purposes of statistical analysis, we divided patients in terms of the number of neoplastic processes into patients with multiple neoplasms with lung cancer being the lung cancer and patients with single lung cancer. In addition, the sex breakdown was taken into account. Thus, we showed that the interval from the development of the first to subsequent cancer in women was significantly longer than in men (8.79 (SD: 8.88); 5.76 (6.52); *p* = 0.015) (Table 1). The time of survival was influenced by a number of factors, for instance, tobacco smoking or genetic predispositions. It is also speculated to depend on physiological differences between the sexes, such as breathing techniques. The lung capacity has its impact on the amounts of carcinogenic compounds accumulated in the lungs. Deeper inhalation of tobacco smoke intensifies the exposure of lungs to the harmful substances it contains. Women find it more difficult to quit smoking and may be exposed to an elevated risk of developing lung cancer at a given level of exposure to tobacco [28,29,30]. Wang et. al, in their 2021 study collating data from all countries around the world, showed that the mortality rate due to lung cancer decreases among men while increasing for women [31]. The data analysis we performed proved that the average survival time of patients with multiple neoplasms of which the last developing one is lung cancer was shorter for women than for men (Figure 2). There are some studies suggesting that women may be more prone to the harmful effects of tobacco smoke than men [32]. In one of the studies, women with a history of 40 pack-years had a three-fold higher risk of developing lung cancer than men with the same pack-year history [33]. According to Raúl Barrera-Rodriguez, women who develop lung cancer are younger and smoke less than men with the same disease [34]. Also, women more often develop adenocarcinoma [34]. These differences may be due to an earlier commencement of the smoking habit, genetic predisposition or different lifestyles. Our study has demonstrated that the most frequent cancer in women with multiple neoplasms is adenocarcinoma and small-cell carcinoma when single neoplasms were diagnosed. 66.96% (n = 1392) of women with multiple neoplasms are smokers. Ossan et al., who conducted a study involving 98 women with a history of small-cell carcinoma, showed that the risk of developing small-cell cancer increases as the frequency of smoking is higher [19]. The authors of the study called Age and Gender Variations in Cancer Diagnostic Intervals in 15 Cancers: Analysis of Data from the UK Clinical Practice Research Datalink revealed that for 6 out of 11 cancers, including lung cancer, the diagnostic period, that is, the time between the manifestation of the first symptoms and making a diagnosis, is longer for women [35]. These authors suggest that women may delay a visit to the doctor when they detect or realize that the symptom they observe is potentially connected with cancer. On the other hand, however, other reports indicate that women are more willing to search for information concerning health [36].

In the paper, we also demonstrated that in the group of smokers, also having included the division into the groups of women and men, patients with single neoplasms lived statistically significantly shorter lives than patients with multiple neoplasms (*p* = 0.000). As already mentioned, most (n = 2174; 94.48%) cases we analyzed were patients with medical histories indicating tobacco dependence. This group was found to comprise 65.01% (n = 1496) of smokers and 29.46% (n = 678) ex-smokers (Table 1). We demonstrated that the survival time of men was longer than that of women among the persons who had quit smoking (Figure 3). The fact that someone stops smoking may have a varied effect on different illnesses linked to smoking. The risk of myocardial infarction due to smoking decreases by 50% in 12 months since the person quit smoking. Smoking cessation inhibits the risk of the development of cancers related to smoking on an experimental level the moment the patient stops smoking, but does not decrease this risk in absolute numbers [37].

In our study on a group of patients hospitalized from 2012 to 2017, the discrepancy between the sexes mentioned above could be seen. The survival times of patients differ depending on the degree of advancement of the neoplastic process, although the prognosis of patients with diagnosed lung cancer is poor. This study proves that the survival time of patients with multiple cancers can be significantly longer than that of patients with a single cancer (Figure 1). According to the data provided by the American Cancer Society, the 5-year-long survival time of patients with non-small-cell lung cancer diagnosed between 2010 and 2016 was 25% on average, while reaching 75% among patients with small-cell cancer [38]. A few years later, in 2016, Smith et al. showed significant differences in tobacco cessation between men and women [39]. It is maintained that women quit smoking less often than men and are less consistent, finding it more difficult to sustain long abstinence periods and thereby resuming the smoking habit more often [40,41].

This finding could be of a secondary nature, resulting from better oncological monitoring, systematically performed imaging tests, and more frequent direct contact with a doctor to whom the patients diagnosed with cancer are submitted. Amer showed that the survivability of patients with multiple cancers is longer than that of patients with a single cancer [42].

Limitation: The Centre for Pulmonary Diseases in Olsztyn is a highly specialized hospital that admits patients with pulmonary illnesses. The patients whose cancer affects other organs than the lungs usually are not hospitalized in this Centre. The data presented in our article were collected from the years 2012 to 2017, which means that a complete comparison of the patients’ survival time is still impossible due to the lack of some statistical data, such as dates of death. Most patients do not live long enough to develop another cancer. Another problem is that the analysis was conducted retrospectively from the data available in the Hospital’s register and the medical data supplemented by the attending physician upon admission to the Ward. It is impossible to verify such data more precisely or to supplement it (most patients are deceased at the time of the analysis). In addition, some of the tests were performed outside the Hospital, in cooperating units (e.g., determination of genetic mutations). These data were delivered to the Hospital with a delay and have not been computerized so far.

## 5. Conclusions

Tobacco smoking is a significant factor influencing the survivability of patients with lung cancer. Smoking cessation results in a longer survival time. Patients with single lung cancer have a poorer prognosis than those with an earlier neoplastic process.

## Figures and Tables

**Table 1:** Tobacco intake and packyears statistics based on gender and/or quantity of neoplasms.

Variable	Total (n = 2301)			Neoplasms								
				Single			Multiple			Neoplasmsa		
	Women	Men	*p*	Women	Men	*p*	Women	Men	*p*	Single	Multiple	*p*
	n; %	n; %		n; %	n; %		n; %	n; %		n; %	n; %	
	724; 31.47	1577; 68.53		654; 31.46	1425; 68.54		70; 31.53	152; 68.47		2079; 90.35	222; 9.65	
Smokers	461; 63.67	1035; 65.63	0.361 ^(1)^	428; 65.44	964; 67.65	0.320 ^(1)^	33; 47.14	71; 46.71	0.952 ^(1)^	1392; 66.96	104; 46.85	0.000 ^(1)^
Ex-smokers	194; 26.80	484; 30.69	0.057 ^(1)^	170; 25.99	411; 28.84	0.179 ^(1)^	24; 34.29	73; 48.03	0.055 ^(1)^	581; 27.95	97; 43.69	0.000 ^(1)^
Non-smokers	69; 9.53	58; 3.68	0.000 ^(1)^	56; 8.56	50; 3.51	0.000 ^(1)^	13; 18.57	8; 5.26	0.002 ^(1)^	106; 5.09	21; 9.46	0.007 ^(1)^
	AVG; SD; M; n	AVG; SD; M; n	*p*	AVG; SD; M; n	AVG; SD; M; n	*p*	AVG; SD; M; n	AVG; SD; M; n	*p*	AVG; SD; M; n	AVG; SD; M; n	*p*
Packyears	32.98; 19.02; 30; 722	39.44; 20.22; 40; 1577	0.000 ^(2)^	33.58; 18.19; 30; 654	39.30; 19.86; 40; 1425	0.000 ^(2)^	27.15; 19.39; 30; 68	40.72; 23.38; 40; 152	0.000 ^(2)^	37.50; 19.73; 40; 2079	36.53; 23.05; 40; 220	0.296 ^(2)^
Packyears smokers	38.78; 16.65; 40; 461	42.41; 19.69; 40; 1035	0.000 ^(2)^	38.84; 16.71 40; 428	42.23; 19; 54 40; 964	0.000 ^(2)^	37.91; 16.15; 40; 33	44.87; 21.70; 40; 71	0.131 ^(2)^	41.19; 18.77; 40; 1392	42.66; 20.29; 40; 104	0.655 ^(2)^
Packyears ex-smokers	30.11; 14.97; 30; 192	37.34; 17.33; 40; 484	0.000 ^(2)^	30.51; 15.22; 30; 170	36.66; 16.28; 40; 411	0.000 ^(2)^	27.05; 12.79; 25; 22	41.15; 22.07; 40; 73	0.004 ^(2)^	34.86; 16.21; 30; 581	37.88; 21.11; 35; 95	0.366 ^(2)^
Packyears non-smokers	2.22; 10.95; 0; 69	3.79; 15.25; 0; 58	0.529 ^(2)^	2.73; 12.11 0; 56	4.40; 16.37; 0; 50	0.581 ^(2)^	0.00; 0.00; 0; 13	0.00; 0.00; 0; 8	0.971 ^(2)^	3.51; 14.23; 0; 106	0.00; 0.00 0; 21	0.231 ^(2)^

Note: ^(1)^ Pearson’s chi-squared test; ^(2)^ non-parametric U Mann–Whitney test.

**Table 2:** Morbidity and survival time with consideration of tobacco intake.

Variable	Total (n = 2301)			Neoplasms								
				Single			Multiple			Neoplasma		
	Women	Men	*p*	Women	Men	*p*	Women	Men	*p*	Single	Multiple	*p*
	AVG; SD; M; n	AVG; SD; M; n		AVG; SD; M; n	AVG; SD; M; n		AVG; SD; M; n	AVG; SD; M; n		AVG; SD; M; n	AVG; SD; M; n	
Age of 1st cancer	63.19; 9.00; 63; 724	64.71; 8.40; 65; 1577	0.000 ^(2)^	63.80; 8.53; 63; 654	64.91; 8.32; 65; 1425	0.007 ^(2)^	57.41; 11.07; 58; 70	62.87; 8.95; 62; 152	0.001 ^(1)^	64.56; 8.40; 64; 2079	61.15; 9.97; 61; 222	0.000 ^(2)^
Age of 2nd cancer	66.20; 7.96; 65; 70	68.63; 7.84; 68; 152	0.015 ^(1)^				66.20; 7.96; 65; 70	68.63; 7.84; 68; 152	0.015 ^(1)^		67.86; 7.94; 67; 222	
Interval between 1st and 2nd cancer (years)							8.79; 8.88; 6; 70	5.76; 6.52; 4;152	0.015 ^(2)^		6.71; 7.46; 4; 222	
General total survival time (years)	65.33; 8.47; 65; 724	66.5; 8.35; 66; 1577	0.003 ^(2)^	65.09; 8.51; 65; 654	66.13; 8.31; 66; 1425	0.012 ^(2)^	67.60; 7.77; 66; 70	69.91; 7.97; 71; 152	0.037 ^(1)^	65.80; 8.39; 66; 2079	69.18; 7.96; 69; 222	0.000 ^(2)^
Smokers’ total survival time (years)	64.49; 7.78; 64.06; 461	65.11; 8.05; 65.00; 1035	0.055 ^(2)^	64.35; 7.71; 63.95; 428	64.98; 8.07; 64.90; 964	0.071 ^(2)^	66.32; 8.48; 66.32; 33	66.94; 7.47; 66.83; 71	0.708 ^(1)^	64.78; 7.97; 64.63; 1392	66.74; 7.77; 66.11; 104	0.014 ^(2)^
Ex-smokers’ survival time (years)	66.14; 8.40; 66.36; 194	68.89; 8.05; 68.53; 484	0.000 ^(2)^	66.05; 8.69; 66.33; 170	68.28; 8.06; 67.85; 411	0.007 ^(2)^	66.82; 6.11; 66.79; 24	72.32; 7.11; 73.22; 73	0.001 ^(1)^	67.63; 8.30; 67.33; 581	70.96; 7.25; 71.51; 97	0.000 ^(2)^
Non-smokers’ total survival time (years)	68.28; 11.74; 68.67; 69	70.17; 10.17; 68.39; 58	0.336 ^(1)^	67.40; 12.40; 67.35; 56	69.65; 10.08; 69.65; 50	0.310 ^(1)^	72.06; 7.54; 72.31; 13	73.44; 10.79; 67.28; 8	0.799 ^(2)^	68.46; 11.37; 68.25; 106	72.58; 8.68; 69.64; 21	0.136 ^(2)^
Survival time since 1st cancer (years)	2.20; 4.04; 0.98; 723	1.85; 3.00; 0.88; 1576	0.075 ^(2)^	1.35; 1.44; 0.85; 653	1.30; 1.50; 0.78; 1424	0.228 ^(2)^	10.19; 8.93; 7.76; 70	7.02; 6.56; 5.12; 152	0.010 ^(2)^	1.31; 1.48; 0.80; 2077	8.02; 7.52; 5.61; 222	0.000 ^(2)^

Note: ^(1)^ parametric Student’s *t*-test; ^(2)^ non-parametric U Mann–Whitney test.

**Table 3:** Total survival time comparison in consideration of tobacco intake—ANOVA and post-hoc tests results.

	Total Survival Time			
	Smoker	Ex-Smoker	Non-Smoker	ANOVA
	AVG; SD; M; n	AVG; SD; M; n	AVG; SD; M; n	*p*
Women ^(2)^	64.49; 7.78; 64.06; 461 ^a B^	66.14; 8.40; 66.36; 194 ^a^	68.28; 11.74; 68.67; 69 ^B^	0.001
Men ^(1)^	65.11; 8.05; 65.00; 1035 ^A B^	68.89; 8.05; 68.53; 484 ^A^	70.17; 10.17; 68.39; 58 ^B^	0.000
Single neoplasm—general ^(2)^	64.78; 7.97; 64.63; 1392 ^A B^	67.63; 8.30; 67.33; 581 ^A^	68.46; 11.37; 68.25; 106 ^B^	0.000
Single neoplasm—women ^(2)^	64.35; 7.71; 63.95; 428 ^a b^	66.05; 8.69; 66.33; 170 ^a^	67.40; 12.40; 67.35; 56 ^b^	0.002
Single neoplasm—men ^(1)^	64.98; 8.07; 64.90; 964 ^A B^	68.28; 8.06; 67.85; 411 ^A^	69.65; 10.08; 69.65; 50 ^B^	0.000
Multiple neoplasms—general ^(2)^	66.74; 7.77; 66.11; 104 ^A b^	70.96; 7.25; 71.51; 97 ^A^	72.58; 8.68; 69.64; 21 ^b^	0.000
Multiple neoplasms—women ^(1)^	66.32; 8.48; 66.32; 33	66.82; 6.11; 66.79; 24	72.06; 7.54; 72.31; 13	0.065
Multiple neoplasms—men ^(2)^	66.94; 7.47; 66.83; 71 ^A^	72.32; 7.11; 73.22; 73 ^A^	73.44; 10.79; 67.28; 8	0.000

^(1)^ Parametric ANOVA. ^(2)^ Non-parametric Kruskall–Wallis ANOVA. ^A^, ^B^—*p* < 0.001; ^a^, ^b^—*p* < 0.05.

## Data Availability

The data presented in this study are available on request from the corresponding author for research purposes only.
